# Developmental Instability and Gene Dysregulation in an Extracted Tetraploid from Hexaploid Wheat

**DOI:** 10.3390/ijms241814037

**Published:** 2023-09-13

**Authors:** Yang Li, Yan Sha, Han Wang, Ruili Lv, Deshi Zhang, Shuhang Li, Chunming Xu, Bao Liu

**Affiliations:** Key Laboratory of Molecular Epigenetics of Ministry of Education (MOE), Northeast Normal University, Changchun 130024, China

**Keywords:** allopolyploidy, subgenome extraction, developmental stability, dysregulated gene expression, co-expression network, homeostasis

## Abstract

The BBAA subgenomes of hexaploid common wheat can be ‘extracted’ to constitute a viable and self-reproducing novel tetraploid wheat, termed extracted tetraploid wheat (ETW). Prior studies have shown ETW manifesting phenotypic abnormalities and alteration in gene expression and epigenetic modifications. No population level investigation has been conducted, leaving the issue unclear regarding whether developmental stability, an essential property evolved in all natural organisms, might have been undermined in ETW. Here, we measured variations in five morphological traits and somatic chromosomal stability in populations of ETW and of its hexaploid donor, a resynthesized hexaploid and a natural tetraploid wheat. We observed phenotypic defects in ETW. Meanwhile, we documented much greater within-population variations in ETW than in the other wheat genotypes, most probably due to disrupted developmental stability in ETW. Also, somatic structural chromosome variations were detected only in ETW. Comparative transcriptome analyses indicated that the disrupted developmental stability of ETW is likely linked to massive dysregulation of genome-wide gene expression rather than to genetic mutations. Population network analysis of gene expression implicated intrinsic connectivity among the variable traits, while gene set enrichment analysis provided possible links between dysregulated gene expression and interlaced trait variation.

## 1. Introduction

Polyploidization or whole genome duplication (WGD) is a driving force in speciation and evolution across the tree of life. Allopolyploidization, which involves WGD of interspecific hybrids, has played a major role in the diversification of plant species [1]. Accumulated studies in diverse plant species have documented that extensive genetic, genomic, transcriptomic, and epigenomic changes with significant phenotypic consequences have either occurred saltationally following allopolyploidization or accrued in the course of post-allopolyploidization genome evolution [1,2,3,4,5,6,7,8,9,10,11,12].

The *Triticum–Aegilops* complex consists of species harboring extensive historical interspecific hybridizations at either homoploid or polyploid level and, thus, represents an excellent system to study genome and organismal evolution associated with hybridization and allopolyploidization [13]. In particular, the complex contains a star-species, i.e., hexaploid common or bread wheat (*Triticum aestivum*), one of the most important staple food crops for human beings, and the speciation of which involves two sequential allopolyploidization events. The first allopolyploidization occurred between an extinct or yet to be discovered diploid *Aegilops* species with a BB genome and diploid wheat *T. urartu* (genome AA) ca. 0.5–0.8 million years ago, which led to the formation of allotetraploid wheat, *T. turgidum* (genome BBAA) [14,15]. Then, single or multiple allohexaploidization event(s) took place between a primarily domesticated form of *T. turgidum* and goat-grass *Aegilops tauschii* (DD genome), leading to the speciation of allohexaploid wheat, *T. aestivum* (genome BBAADD) less than 10,000 years ago [15,16].

The A, B, and D subgenomes of common wheat have largely retained the original structures of their diploid progenitor genomes, with only a few intra-chromosomal inversions and inter-chromosome translocations [17]. Because of this high degree of subgenome integrity, the BBAA subgenomes of hexaploid wheat were can be “extracted” out by hybridizing this wheat with a tetraploid *durum* wheat, and then we can recurrently backcross the F1 pentaploid hybrids to the hexaploid wheat donor to eventually eliminate the DD genome and form a new tetraploid wheat (BBAA) known as “extracted tetraploid wheat” (ETW) [18]. ETW provides a unique opportunity to assess the extent to which the BBAA subgenomes have been irreversibly sculpted, both in the process of allohexaploidization and during the course of its coexistence with the DD subgenome in hexaploid wheat evolution, domestication, and improvement.

Prior studies have identified severe phenotypic anomalies in ETW, including dwarfed stature, decreased number of tillers, more compacted spikes, and reduced fertility, although it maintains meiosis stability [18,19]. Phenotypic normalcy could be fully restored by reintroducing the DD genome of *Ae. tauschii* to ETW, with the resynthesized allohexaploid wheat (XX329) showing normal growth/development and high fertility [18,19]. This indicates the indispensable dependency of the BBAA subgenomes of hexaploid wheat on the presence of the DD subgenome for normal functionality.

Further studies in ETW have also revealed its significant changes in gene expression relative to both its hexaploid wheat donor and natural tetraploid *durum* wheat [19]. In particular, it was found that downregulated genes in ETW are enriched in gene ontology (GO) terms related to chloroplast/plastid functions, suggesting compromised photosynthesis in ETW [19]. Additionally, two recent studies have investigated the changes in epigenetic modifications during the ploidy transition processes [20,21]. One study showed that DNA methylation levels in endosperm, especially in the CHG context, are significantly decreased in ETW compared to its hexaploid wheat donor, but the demethylated regions in ETW were remethylated in the resynthesized hexaploid wheat by reintroducing the DD subgenome [20]. The other study reported overall stability of two histone modification markers, H3K4me3 and H3K27me3, during the ploidy transitions, but a subset of genes showed higher histone modification levels in ETW than in its hexaploid donor, which were also restored to normal levels in the resynthesized hexaploid wheat [21]. Both changes in DNA methylation and histone modifications in ETW have been found to be associated with changes in gene expression [20,21].

Developmental stability, i.e., the capacity to maintain phenotypic normalcy and consistency, thereby ensuring a robust fitness in the face of cryptic genetic variation and mild environmental perturbation, is an intrinsic as well as essential property evolved in all organisms [22]. Conceivably, maintaining cellular physiological homeostasis enabled by tight regulation of gene expression is likely a major mechanistic underpinning of developmental stability. In light of this consideration, the foregoing described substantial alterations in both gene expression and epigenetic modifications in the ETW promoted us to ask the question as to whether developmental stability or phenotypic consistency (canalization) might have been undermined in this novel allotetraploid wheat. Answering this question may provide novel insights into the functional interdependency of subgenomes in an established allopolyploid organism and inform future efforts to artificially construct new allopolyploid crops.

In this study, we conducted a comprehensive assay of phenotypic variation in several major phenotypic traits between ETW and its hexaploid wheat donor (TAA10), a resynthesized hexaploid wheat (XX329) using ETW and *Ae. tauschii* as hybridization parents, and a natural allotetraploid *durum* wheat (TTR13) [18]. We assessed the phenotypic stability of ETW at the population level and interrogated the relationships between different traits. We performed RNAseq-based transcriptome profiling to investigate the patterns of gene expression in ETW relative to the other relevant wheat genotypes. Furthermore, we analyzed co-expression networks of genes associated with the phenotypic instabilities of the ETW population, aiming at identifying potential regulatory gene sets involved in the trait variations. By employing the single-sample gene set enrichment analysis (ssGSEA) method, we explored GO terms with significant differences in gene expression distribution between the population ETW and those of the two hexaploid wheat genotypes (TAA10 and XX329), and further investigated their networks using CytoScape. Through these integrated analyses and comparisons, we identified several biological processes and molecular functional gene sets that may contribute to the disruption of developmental stability in ETW at the population level.

## 2. Results

### 2.1. Phenotypic Defects and Disrupted Developmental Stability in the Extracted Tetraploid Wheat

**Figure 1:**
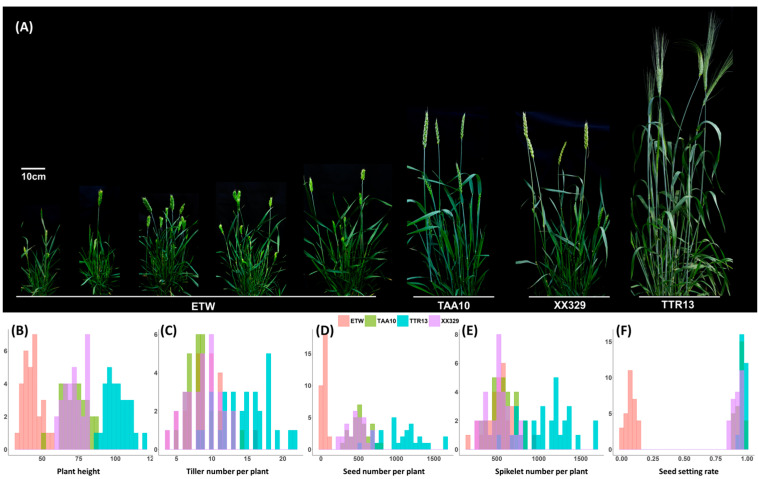
Greater phenotypic variation among individuals of the extracted tetraploid wheat (ETW) relative to other wheat genotypes. (**A**) Overall plant architecture of five representative plant individual of ETW along with one individual of each of its hexaploid donors (TAA10), a resynthesized allohexaploid wheat (XX329), and a tetraploid durum wheat (TTR13). (**B**–**F**) Histograms depicting variations in each of the five traits of the four wheat genotypes.

By measuring and quantifying 5 morphological traits in various wheat genotypes (30 individuals per genotype), we observed pronounced phenotypic deviations in the extracted tetraploid wheat (ETW) compared to other genotypes grown under the same regular field conditions. Phenotypic comparisons among different wheat genotypes revealed significant disparities between ETW and hexaploid wheat (TAA10 and XX329) as well as tetraploid wheat (TTR13) across multiple traits, including notably reduced seed setting, and number of seeds per plant (Mann–Whitney test *p*-value ≤ 6.03 × 10^−11^; Figure 1A,B,D,F; Tables S1 and S2). However, tiller number per plant and spikelet number per plant in ETW exhibited no significant differences when compared to its hexaploid donor (TAA10) and the resynthesized hexaploid (XX329) (Mann–Whitney test *p*-value ≥ 0.35; Figure 1; Tables S1 and S2). Nevertheless, these traits did show significant differences compared to the tetraploid durum wheat (TTR13) (Mann–Whitney test *p*-value ≤ 1.78 × 10^−7^; Figure 1; Tables S1 and S2). These results suggest that not all traits in ETW were equally prone to defects; rather, some traits exhibited greater stability and closely retained the characteristics of their hexaploid wheat donor and the resynthesized hexaploid wheat, implying a higher degree of canalization for these specific traits.

Much greater variations were observed in the extracted tetraploid wheat (ETW) population than in those of other wheat genotypes. The coefficient of variations (CVs) for all five traits in the ETW population were greater compared to the other genotypes (Table S1). In particular, the CVs for seed setting rate (52.78%) and seed number per plant (64.82%) were significantly higher than those of the other four genotypes, in which the CV for seed setting ranged from 1.86% to 3.78%, and the CV for seed number per plant ranged from 24.02% to 27.93% (*p*-value < 0.001; Tables S1 and S4). Regarding the other traits, while the CVs observed in ETW were generally higher than those observed in the other genotypes, statistical significance was not reached, except for plant height. Here, the CV for plant height in ETW exhibited a statistically higher value than that of TTR13 (*p*-value = 0.0036; Table S4). These results suggest that, in comparison to the other four wheat genotypes, ETW exhibited apparent defects and variations in phenotypes.

**Figure 2:**
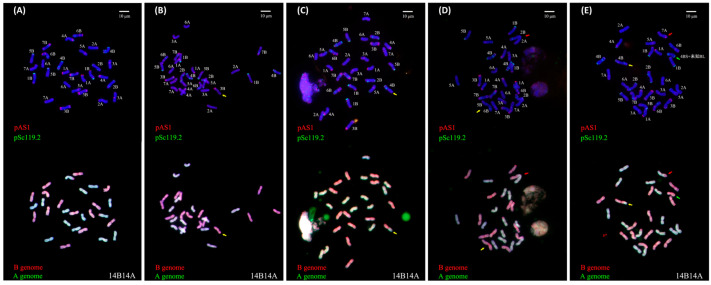
Representative metaphases of root-tip cells of ETW showing karyotype instability that is manifested by structural chromosome variations (SCVs) detected by the combined use of FISH and GISH. (**A**) Euploid individuals without SCVs. (**B**) Loss of the short arm of chromosome 3B (indicated by a yellow arrow). (**C**) Replacement of the short arm of chromosome 4B with an unknown A-subgenome chromosome arm (indicated by a yellow arrow). (**D**) Fusion of the short arm of chromosome 2B with the long arm of chromosome 2A (indicated by a red arrow), fusion of the short arm of chromosome 5B with the short arm of chromosome 6B, and loss of the short arms of chromosomes 5B and 6B (indicated by a yellow arrow). (**E**) Simultaneous occurrence of a translocation between the short arms of chromosomes 7A and 7B (indicated by a red arrow), a translocation between chromosome 4B and an unknown subgenome A chromosome segment (indicated by a yellow arrow), and loss of one chromosome 7B and gain of a fused chromosome involving the short arm of chromosome 4B and an unknown subgenome B chromosome fragment (indicated by a green arrow). In FISH, pAS1 (red) and pSc119.2 (green) were used as probes. In GISH, DNA from *T. urartu* (AA) (yellow-green coloration) and *Ae. bicornis* (SS) (pink coloration) were used as probes.

To test whether the developmental instability in ETW may lead to a more severe consequence, that is, somatic karyotype instability, we examined metaphase chromosomes of root-tip cells of ETW and of the other three wheat genotypes (as controls) by the combined use of florescence in situ hybridization (FISH) and genomic in situ hybridization (GISH), which enables unequivocal identification of all chromosomes of both tetraploid and hexaploid wheat [19]. We found that root-tip cells in 25.9% (21 out of 81) of the studied individuals of ETW did indeed show structural chromosomal variations (SCVs) (Figure 2B–E), whereas all the other three genotypes have stable karyotypes as expected. Notably, these SCVs did not stem from meiotic irregularities but from aberrant mitosis, because no plant showed the same type of SCV in the multiple metaphases analysis, even of the same root-tip. Instead, several different types of SCVs could be detected in different metaphases, which included loss of chromosomal fragments, translocations, and fusion between different chromosomes (Figure 2A–E). This unexpected cytogenetic result indicates that genomic stability in somatic cells of ETW was also undermined, underscoring the severeness of its developmental instability during ontogenesis.

### 2.2. Disrupted Developmental Stability of ETW Is Not Due to Molecular Level Genetic Alterations

**Figure 3:**
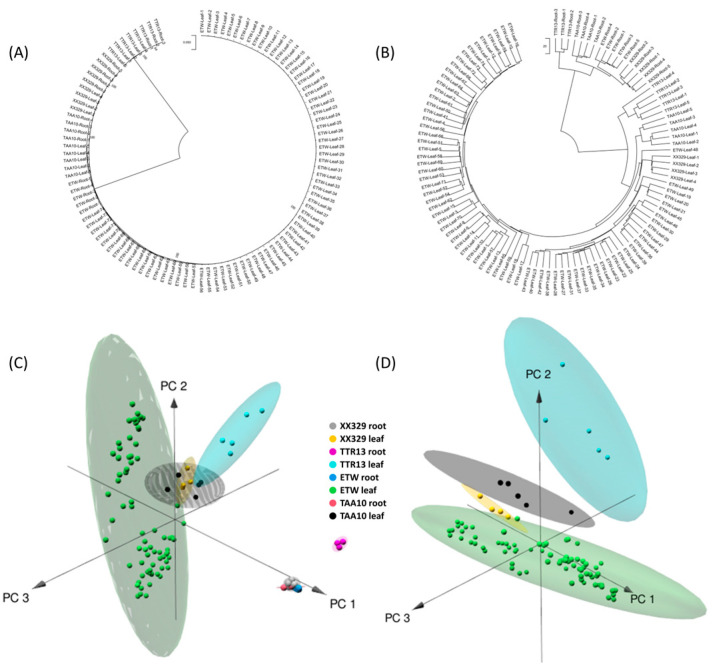
Genetic and transcriptomic (genome-wide gene expression) relationships among the studied samples of four wheat genotypes. (**A**) Phylogenic relationship among the samples. (**B**) Cluster dendrogram of all that samples based on gene expression values. (**C**) A PCA plot for the root-tip samples. (**D**) A PCA plot for the leaf samples.

In order to gain some insights into the molecular basis underlying the developmental instability in ETW, we conducted RNAseq-based transcriptome profiling on the leaf tissue of a total of 74 plant individuals of ETW, along with 5 individuals of TAA10 (the hexaploid donor to ETW), 5 individuals of TTR13 (a *durum* tetraploid wheat), and 4 individuals of XX329 (a resynthesized hexaploid wheat with ETW as the maternal parent). Additionally, we analyzed transcriptomes of the root-tip tissue of 5 mixed ETW pools (each pool containing root-tips taken from 10 randomly chosen plants), 4 TAA10 pools, 5 TTR13 pools, and 5 XX329 pools. First, to test whether the developmental instability of ETW was linked to molecular level genetic alterations, we performed phylogenetic analysis based on single nucleotide polymorphisms (SNPs) of expressed genes identified based on the RNAseq data of the BBAA genomes (of the two tetraploid wheat genotypes) and subgenomes (of the two hexaploid wheat genotypes) for all samples. The results of the generated phylogenetic tree indicated that all ETW samples were clustered together with all samples of TAA10 and XX329 (Figure 3A), indicating the phenotypic variations (reflection of developmental instability) among the ETW individuals was not due to molecular-level genetic changes. Moreover, the hierarchical cluster analysis based on the Euclidean distance of gene expression data revealed distinct clustering of leaf and root-tip samples, indicating greater expression differences between tissue types than between genotypes (Figure 3B). Specifically, in both leaf and root-tip tissues, the ETW samples clustered closely with XX329 initially and then formed a separate branch from TAA10, which was consistent with the relationships of genomic origin/background among the genotypes (Figure 3B). The distribution of samples based on the three principal components of expression data further supported the hierarchical cluster results (Figure 2C). Notably, we observed that the ETW leaf samples exhibited a higher degree of dispersion compared to the other genotypes in the PCA plot (Figure 2D), suggesting diverse regulations of gene expression in the ETW samples, as detailed below.

### 2.3. Phenotypic Defects of ETW Is Likely Linked to Massive Dysregulation of Genome-Wide Gene Expression

We analyzed differentially expressed genes (DEGs) in each of all the pairwise comparisons involving all the four wheat genotypes in both leaf and root-tip tissues. The results indicated a much greater number of DEGs in leaf tissue (ranging from 5506 to 15,337) than in root-tip tissue (ranging from 440 to 4575) in all genotype pairs (Table 1). Of note, the comparisons between ETW and the two hexaploid wheat genotypes (XX329 and TAA10) showed opposite trends in the relative numbers of up- and downregulated DEGs in the two tissues. In the leaf, the numbers of upregulated DEGs in both ETW vs. XX329 and ETW vs. TAA10 (ranging from 5132 to 6138) were significantly higher than the numbers of downregulated genes (ranging from 3609 to 4429) (binomial test *p*-value ≤ 6.84 × 10^−13^). In contrast, in the root-tip, there were significantly more downregulated genes in both ETW vs. TAA10 and ETW vs. XX329 (ranging from 1521 to 1715) than upregulated genes (ranging from 537 to 533) (binomial test *p*-value < 2.2 × 10^−16^) (Table 1). As expected, the XX329 vs. TAA10 comparisons showed the lowest numbers of DEGs in both the leaf (5506) and root-tip (440), as expected given that the only difference between them is the DD subgenome, one being the original of hexaploid wheat (TAA10) and one being of an accession of *Ae. tauschii* [18].

Further investigation of the pairwise DEGs revealed that in the leaf, a substantial number of upregulated DEGs (2207) were shared between ETW and TAA10 and between ETW and XX329, while the number of shared downregulated genes (1039) was relatively small (Figure S1A). Conversely, in root-tip, most of the downregulated DEGs (1025) were shared between ETW and TAA10 and between ETW and XX329, whereas only 171 upregulated DEGs were shared between the two pairwise comparisons (Figure S1B). These results highlight the tissue-specific dysregulation of gene expression in ETW relative to its allohexaploid donor and the resynthesized allohexaploid, while the shared DEGs may provide valuable hints towards delineation of potential common regulatory mechanisms underlying the more strongly canalized traits (developmentally stable) in allohexaploid wheat.

Mapping of the up- and downregulated DEGs to each of the 14 BBAA chromosomes revealed a broadly even distribution along most chromosomes. However, some chromosomes showed higher numbers of up- or downregulated DEGs relative to the other chromosomes (Figures S2 and S3). Interestingly, in both leaf and root-tip tissues, ETW vs. TAA10 showed significantly higher numbers of up- than downregulated genes on Chromosome 1A (binomial test *p*-value ≤ 9.90 × 10^−14^, Figures S2 and S3). In contrast, in root-tip tissue, apart from Chromosome 1A, all of the other chromosomes exhibited a significantly higher numbers of down- than upregulated genes in ETW vs. TAA10 (binomial test *p*-value ≤ 5.05 × 10^−6^, Figure S3). Also, in the pairwise comparisons of ETW vs. TAA10 and XX329 vs. TAA10, we noticed a distinctive pattern with upregulated DEGs showing a continuous and dense distribution at the end of Chromosome 1A in both tissues (Figure S4). Similarly, in the TAA10 vs. TTR13 comparison, the downregulated DEGs exhibited a continuous and dense distribution in the same region. In contrast, when comparing ETW vs. TTR13, XX329 vs. TTR13, and ETW vs. XX329, we did not observe significant enrichment of either up- or downregulated DEGs in any specific chromosomal regions (Figure S4). This intriguing observation may suggest that expression of genes residing on this particular chromosomal region have been irreversibly impacted by the ploidy transition, probably due to heritable epigenetic changes.

**Figure 4:**
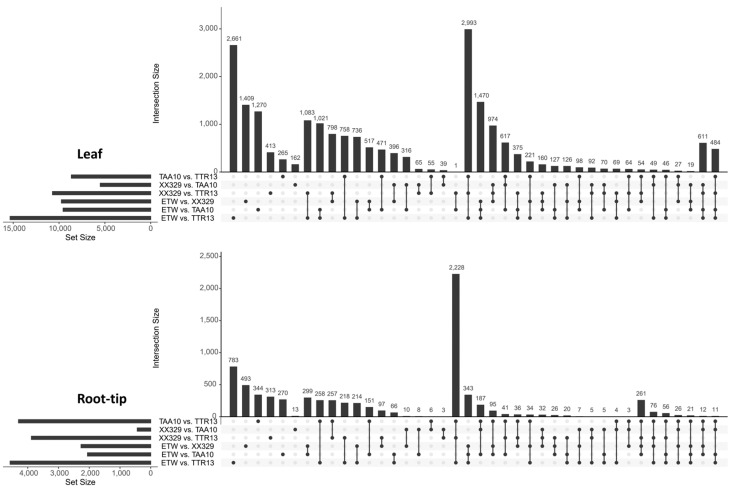
Upset plots of DEGs among different inter-genotypic pairwise comparisons in two tissues, leaf (**upper**) and root-tip (**lower**). Left, bar-plots showing the total numbers of DEGs in each comparison. Right, bar-plots showing the numbers of pair-specific and overlapped DEGs.

Next, we utilized upset plots to explore the relationships among DEGs in the various inter-genotypic pairwise comparisons (Figure 4). Results revealed that the ETW vs. TTR13 comparison showed the highest number of pair-specific DEGs which were non-overlapping with other comparisons in both the leaf (2661) and root-tip (783) (Figure 4). Additionally, the number of overlapping DEGs among ETW vs. TTR13, XX329 vs. TTR13, and TAA10 vs. TTR13 was the highest in both the leaf (2993) and root-tip (2228), indicating a sizable proportion of the transcriptomes of ETW, its hexaploid donor (TAA10) and the resynthesized hexaploid wheat (XX329) were commonly different from the tetraploid *durum* wheat (Figure 4).

To gain further insights into the functional implications of DEGs among the various wheat genotypes, we conducted a gene ontology (GO) and Pfam enrichment analysis. In leaf tissue, several GO terms involved in protein heterodimerization activity, polysaccharide binding, photosynthesis light harvesting, metabolic process, ATP-binding, and the nucleosome were significantly overrepresented in DEGs of at least two of the three pairwise comparisons, namely ETW vs. TAA10, ETW vs. XX329, and ETW vs. TTR13 (Figure S3). In the root-tip, DEGs in both of the ETW vs. TAA10 and ETW vs. XX329 pairwise comparisons displayed highly similar enriched GO terms that were associated with DNA-binding transcription factor activity, regulation of DNA-templated transcription, transporter activity, response to stress, response to water, embryo development, photosynthesis, ribonuclease T2 activity and so on (Figure S5, Supplementary Materials). For overrepresented Pfam terms, in leaf tissue, the Hsp20/alpha crystalline family and wall-associated receptor kinase galacturonan-binding term were found to be significant in all comparisons between ETW and the other genotypes; meanwhile, core histone H2A/H2B/H3/H4 term and chlorophyll A-B binding protein term were overrepresented in ETW vs. TAA10 and ETW vs. TTR13 but was not in ETW vs. XX329. In root-tip tissue, DEGs in both of the ETW vs. TAA10 and ETW vs. XX329 pairwise comparisons displayed highly similar enriched Pfam terms, such as dehydrin and WRKY DNA-binding, that were in line with the overrepresented GO terms (Figure S6, Supplementary Materials).

### 2.4. Disrupted Developmental Stabilities Likely Link to Co-Expression Networks in the ETW Population

**Figure 5:**
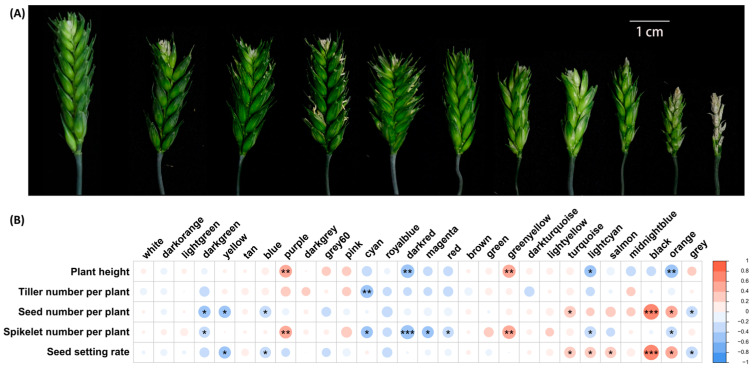
Phenotypic variation and correlated co-expression modules in the ETW population. (**A**) Representative spike phenotypes of the ETW individuals grown under greenhouse conditions. (**B**) Correlation between modules and traits in the ETW population. Correlation coefficients are denoted by colors. *, ** and *** denote *p*-values < 0.05, < 0.01 and < 0.001, respectively.

We examined the phenotypes of 35 individual plants of the ETW population grown under greenhouse condition, which were among those used for RNAseq (Figure 5A, Table S3). We found substantial phenotypic diversity among these individuals of the ETW population. Moreover, different traits showed significant correlations between each other (Table 2). Specifically, we found that plant height, tiller number per plant, and spikelet number per plant exhibited significant positive correlations with each other, while seed setting showed a significant correlation with number of seeds per plant (Table 2). To further explore the factors contributing to the phenotypic variation in the ETW population, we constructed gene co-expression networks using the transcriptome data of the 35 individuals.

A total of 27 co-expression network modules, along with a module of ungrouped genes (module “grey”), were identified, indicating that most dysregulated genes in ETW individuals were intrinsically linked. Next, we examined the relationships between each module and different traits in the 35 individuals. Our analysis revealed significant associations between all traits and at least one module (*p*-value < 0.05) (Figure 5B, Tables S5 and S6). We identified seven modules significantly associated with the number of seeds per plant (*p*-value < 0.05) and eight modules significantly associated with seed setting (*p*-value < 0.05) (Figure 5B, Tables S5 and S6). Remarkably, the majority of modules that showed significant association with both seed setting and number of seeds per plant were found to be the same (Figure 5B, Tables S5 and S6). Specifically, six modules displayed significant correlations with both traits, suggesting that they were underpinned by the same or similar genetic mechanisms. Notably, module “black” exhibited the highest correlation with both seed setting and number of seeds per plant (Figure 5B, Tables S5 and S6). Additionally, we identified five significant modules that were associated with spikelet number per plant, two with plant height, and one with tiller number per plant, suggesting the involvement of distinct genetic regulatory networks in these traits (Figure 5B, Tables S5 and S6).

We further identified hub genes (module membership > 0.8 and gene significance > 0.3) within the modules that exhibited the highest correlation with each trait. Functional analysis of hub genes within the module (module ”black”) that showed the strongest correlation with seed setting and seed number per plant identified several transcription factor-coding genes, such as MYB and AP2-domain genes. Additionally, several hub genes were found to be homologs of *Arabidopsis thaliana* genes known to be involved in the regulation of these traits (Supplementary Materials). For instance, we found that *TraesCS2A03G1072000*, a hub gene in module “black”, is a homolog of the *Arabidopsis FERONIA* (*FER*) gene that mediates male–female gametophyte interactions during pollen tube reception [23]. Another gene, *TraesCS7B03G0836200*, identified as a hub gene in the same module, corresponded to *Arabidopsis OXOPHYTODIENOATE-REDUCTASE 3* (*OPR3*), mutations which lead to male sterility and defective pollen dehiscence [24]. Furthermore, *TraesCS2A03G0409800*, another hub gene in module “black”, was found to be a homolog of the *Arabidopsis PAL1* gene that is known to be involved in pollen production and fertility [25]. Additionally, *TraesCS2B03G0677700*, a homolog of the *Arabidopsis HUA ENHANCER 1* (*HEN1*) gene was among the hub genes in module “darkred”, associated with plant height and number of spikelet per plant. In *Arabidopsis*, *HEN1* plays a role in specifying reproductive organ identities and shares *AGAMOUS*’s non-homeotic function in controlling floral determinacy [26]. Mutations in *HEN1 (hen1)* result in a range of developmental defects, including reduced leaf size, diminished plant height, and corymb-like inflorescences [26].

### 2.5. Dysregulated Functional Gene Sets in the ETW Population Relative to the Other Wheat Genotypes

**Figure 6:**
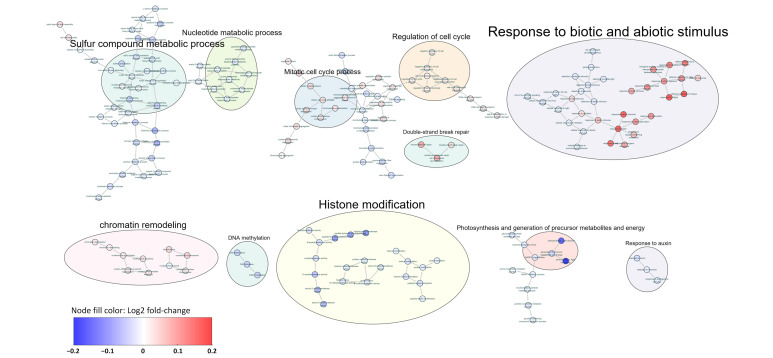
Network depicting significantly altered GO terms in the ETW population. Each node within the network represents a specific GO term. Nodes linked by edges indicate relationships characterized as “is_a”, “negatively_regulates”, “positively_regulates”, or “regulates”. Related terms were manually clustered and annotated by circles. The log2 transferred fold-change of ssGSEA sores between the population of ETW and those of hexaploids are shown in different colors.

We employed the single-sample gene set enrichment analysis (ssGSEA) method [27] to generate projected values for the overall expression distribution of genes associated with GO terms in each leaf sample. Then, we used individuals of TAA10 and XX329 as controls to curate differentially expressed GO terms in the ETW population (*q*-value < 0.05, Supplementary Materials). By analyzing the GO networks, we observed significant differences in the overall expression of GO terms related to epigenetic modifications in the ETW population compared to the hexaploids (Figure 6). Notably, GO terms associated with histone modification and DNA methylation were coincidently downregulated in the ETW population, while almost all terms related to chromatin remodeling, such as condensed chromatin and protein–DNA complex subunit organization, were upregulated (Figure 6). Furthermore, GO terms related to mitotic cell cycle process and regulation of cell cycle were significantly upregulated in the ETW population (Figure 6), consistent with the observed aberrant mitosis (Figure 2). Several GO terms associated with metabolic processes, including the nucleotide metabolic process, sulfur compound metabolic process, and response to auxin, were significantly downregulated in the ETW population (Figure 6). Moreover, a considerable number of gene sets related to the response to biotic and abiotic stimuli exhibited significant differential expression in the ETW population compared to the hexaploid wheat genotypes (Figure 6). Specifically, stress-responsive functional genes, such as those involved in response to water deprivation and defense against fungi, showed significant overall upregulation in the ETW population (Figure 6). Additionally, terms related to photosynthesis and generation of precursor metabolites and energy were significantly downregulated in the ETW population (Figure 6), which accords with the previously observed phenotypes of ETW showing compromised photosynthesis and reduced biomass [18,19]. We repeated the analysis by using individuals of all of the other three genotypes, namely TAA10, XX329 and TTR13, as controls, and obtained similar GO terms that were significantly changed in the ETW population (Figure S7, Supplementary Materials).

## 3. Discussion

Common wheat (*T. aestivum*) is a young allohexaploid species containing three subgenomes (BBAADD) donated by three diploid progenitor species of the *Triticum–Aegilops* complex [13]. As early as 1966, E.R. Kerber experimentally demonstrated that the BBAA subgenomes of common wheat still retain sufficient integrity such that they can be ‘extracted’ to constitute a viable and self-reproducing tetraploid wheat, termed extracted tetraploid wheat (ETW) [18]. Nonetheless, follow-up studies have shown that ETW is actually ‘sub-functional’ and manifests phenotypic defects in multiple traits as well as having marked differences in gene expression and epigenetic modifications compared with natural tetraploid and hexaploid wheats [19,20,21,28]. Of note, all these prior studies have treated ETW as a uniform pure-line genotype and did not pay attention to its possible inter-individual variation in any aspect. This renders the issue unclear with respect to whether developmental stability, an essential property evolved in all natural organisms [22], might have been undermined in ETW given that its formation exclusively depends on artificial manipulations.

We have addressed the above-raised issue in this study. Through comprehensive analyses of phenotypic variation in five representative morphological traits reflecting growth and ontogenic development at the population level in ETW in comparison with its allohexaploid wheat donor, a resynthesized allohexaploid wheat with ETW as its maternal parent [18], and a natural tetraploid *durum* wheat, we show that much greater phenotypic variation occurred in ETW than in the other wheat genotypes. Moreover, ETW also manifested diverse chromosomal structural variations in somatic cells, a phenomenon that usually does not occur in plants under natural conditions. These results, together with the previous findings that epigenetic modifications (including both DNA methylation and histone modifications) and gene expression were substantially altered in ETW, promoted us to suspect that the within-population phenotypic variations were most likely due to disrupted developmental stability. This is because the capacity to maintain a robust cellular physiological homeostasis that conceivably entails tight regulation of gene expression and epigenetic state are likely essential to maintaining developmental stability.

Indeed, our RNAseq-based transcriptome profiling data involving all possible pairwise comparisons between ETW and each of the other three wheat genotypes, i.e., the hexaploid donor (TAA10) to ETW, a resynthesized hexaploid (XX329) using ETW as maternal parent, and a natural tetraploid *durum* wheat (TTR13), unraveled extensive dysregulation of a large number of genes. In particular, given that only genes mapped to the BBAA genomes were considered, and the two hexaploids (TAA10 and XX329) virtually contain the same BBAA as ETW, DEGs in the ETW vs. TAA10 and ETW vs. XX329 comparisons clearly indicate *trans*-regulation by the DD subgenome plus those impacted by the altered epigenetic modifications [19,20,21,28]. Unfortunately, because all kinds of epigenetic modifications hitherto investigated were found to be reverted upon reintroduction of the DD subgenome in XX329, it is not possible to separate the two factors. Nonetheless, our results for this study establish that after ca. 10,000 years of co-evolution in hexaploid wheat, the BBAA subgenomes, albeit still largely intact, have lost an essential property, i.e., developmental stability or canalization, suggesting inter-dependency and essential collaboration of subgenomes in an established allopolyploid species for normal organismal functionality.

Our transcriptome analyses, which included two tissues, namely leaf and root-tip, also indicated that the effects of ploidy transition and subgenome extraction on gene expression bear tissue specificity. Furthermore, we identified a genomic region on chromosome 1A that displayed a concentrated upregulated DEGs, and which were common in the ETW vs. TAA10 and XX329 vs. TAA10 comparisons. Likewise, in the TAA10 vs. TTR13 comparison, the downregulated DEGs also exhibited a continuous and dense distribution within the same region (Figure S4). These findings strongly suggest that many genes located in this specific chromosomal region have been irreversibly affected by the ploidy transitions, leading to distinct gene expression patterns in both ETW and the resynthesized hexaploid XX329 compared to the donor hexaploid TAA10. These effects could be attributed to either genetic or epigenetic changes, which warrants further studies to delineate.

Our study uncovered significant correlations between or among the different traits in the ETW population. Through the analysis of the gene co-expression network, we identified co-expression modules associated with specific traits. In each trait’s most correlated module, we found hub genes functionally related to the regulation of the corresponding phenotypic variations, suggesting that dysregulation of these genes may contribute to the phenotypic variation observed in the ETW population due to undermined developmental stability. Moreover, by comparing ssGSEA scores of GO terms between genotypes, we identified functional gene sets with significant differences in expression profile between ETW and the two hexaploid wheat genotypes. Analyzing the network relationships among these GO terms, we further found that the downregulated GO terms were related to photosynthesis, consistent with previous findings of reduced photosynthesis capacity in ETW [19]. We also found that the upregulated GO terms were related to stress responses while downregulated terms were related to metabolism, which may be associated with the previously observed much stronger accumulation of several kinds metabolites in ETW [28].

Epigenetic regulation plays a pivotal role in both development and stress responses in plants [29]. Previous studies have uncovered significant alterations in DNA methylation and H3K4me3 and H3K27me3 histone modifications in ETW [20,21]. In this study, we further identified widespread differences in GO terms related to DNA methylation, histone modifications, and chromatin remodeling in ETW relative to the other wheat genotypes, which may further suggest substantial perturbation in epigenetic regulatory mechanisms in ETW. The dysregulation of genes involved in these epigenetic modification processes could be a major underlying factor contributing to the compromised developmental stability in ETW at the population level, which also warrants further empirical investigations.

## 4. Materials and Methods

### 4.1. Growth Conditions, RNA Extraction and Sequencing

All genotypes, including the “extracted tetraploid wheat” (ETW), its hexaploid donor (TAA10), the resynthesized hexaploid (XX329), and a natural tetraploid durum wheat (TTR13), were grown in the field to assess agronomic traits. Five specific traits, namely plant height (cm), number of tillers, number of spikelets per plant, seeds per plant, and seed setting rate, were evaluated for 30 individuals of each genotype. The descriptive statistics of each trait were calculated using R (version 4.1.3). The tests for significant differences in coefficients of variation (CVs) between genotypes were performed using a method proposed by Krishnamoorthy, K. and Lee, M. 2014 in the R package “cvequality” [30]. For RNAseq analysis, all genotypes were cultivated under controlled conditions in a greenhouse. Young leaves from 74 ETW individuals, 5 TAA10 individuals, 5 TTR13 individuals, and 4 XX329 individuals were collected separately. Additionally, 5 root samples from mixed ETW individuals, 4 root samples from mixed TAA10 individuals, 3 root samples from mixed TTR13 individuals, and 5 root samples from mixed XX329 individuals were also collected. Among the 74 ETW individuals used for RNAseq, the phenotype of 35 individuals was measured. Total RNA extraction was performed using TriPure reagents (Roche Diagnostics GmbH, Mannheim, Germany) following the manufacturer’s instructions for each sample. Subsequently, the RNAseq libraries were constructed and sequenced on the MGISEQ-2000 at BGI, generating 10G bases of clean data for each sample.

### 4.2. Fluorescence In Situ Hybridization (FISH) and Genomic In Situ Hybridization (GISH)

The protocols for fluorescence in situ hybridization (FISH) were performed following the methodologies described by Han et al. [31] and Kato et al. [32], with minor modifications. In brief, for FISH analysis, two repetitive DNA sequences, namely pSc119.2 and pAS1, were labeled with Alexa Fluor 488-5-dUTP (green coloration, Thermo Fisher Scientific, Eugene, Oregon, U.S.) and Texas Red-5-dCTP (red coloration, Cytiva, Little Chalfont, Buckinghamshire, U.K.), respectively. These labeled probes were then sequentially hybridized to the same set of slides. This dual-probe FISH approach enabled the identification of all 7 homologous chromosome pairs. Additionally, genomic in situ hybridization (GISH) was performed using genomic DNA from *T. urartu* (AA) and *Ae. bicornis* (SS), which were labeled by nick translation with Alexa Fluor 488-5-dUTP and Texas Red-5-dCTP, respectively. Metaphase chromosome spreads were prepared following the methodology outlined by Kato et al. [32]. For the slide denaturation, hybridization, and washing steps, we followed the recommendations provided by the manufacturer (Invitrogen; no. C11397) to ensure optimal conditions for FISH and GISH experiments. Subsequently, the slides were examined using an Olympus BX61 fluorescence microscope, and digital images were captured using the Olympus IPP software package.

### 4.3. RNAseq Data Processing, Mapping, and Identifying Differentially Expressed Genes

RNAseq data were subjected to quality filtering using Trimmomatic. The resulting clean data were then aligned to the hexaploid wheat reference genome (IWGSC2.1) using HISAT2 (version 2.1.0) [33] with the default settings. Subsequently, featureCounts (version 2.0.1) [34] was employed to count the number of uniquely mapped reads for each gene. For subsequent analysis, only high-confidence genes from the A- and B-subgenomes were considered. To further refine the dataset, gene expression data were filtered, and genes with average read counts greater than 10 and less than 5000 were retained for downstream analysis. Differentially expressed genes (DEGs) between different genotypes were identified using DESeq2 (Wald test) [35] with a significance threshold of adjusted *p*-value < 0.05. The normalized read counts of the identified DEGs were log2-transformed and scaled, and based on these transformed expression values, Euclidean distances among samples were calculated. Hierarchical cluster analysis was performed using the “hcluster” function in R (version 4.2.0) with the “average” method, allowing us to explore patterns of similarity among the samples.

### 4.4. Variant Calling and Phylogenetic Analysis

The aligned RNAseq data were used for variant calling. Variants were jointly called and genotyped using GATK (version 4.1.4.0) [36]. The biallelic SNPs were extracted using VCFtools [37] with a setting of “--min-alleles 2 --max-alleles 2 --minGQ 20 --minDP 3 --max-missing 1”. The variants were transformed to fasta sequences for all samples using “vcf2phylip.py” (https://github.com/edgardomortiz/vcf2phylip, accessed on 22 July 2023). The sequences were aligned and phylogenetic tree was constructed using Neighbor-Joint method in MEGA-X [38].

### 4.5. GO and Pfam Enrichment Analysis of DEGs

DEGs between genotypes were used for gene ontology and Pfam enrichment analysis. Terms containing less than 5 expressed genes were removed from further analysis. A one-tail hypergeometric test was used to test whether a term was overrepresented in DEGs. The raw *p*-values were adjusted using the FDR method and only terms whose adjusted *p*-value less than 0.05 were classified as significantly overrepresented.

### 4.6. Gene Co-Expression Network Analysis of ETW Leaf Samples

The weighted correlation network analysis was carried out using a WGCNA package with gene expression data (log2-transferred FPKM) of the 35 ETW leaf samples [39]. For network construction, most parameters were set to default, although the power and TOMtype were set to 14 and “unsigned”, respectively. The module–trait association analysis was performed to identified significantly associated (*p*-value < 0.05) modules for each trait. Hub genes of the most significant module of each trait were identified using the cutoff of module membership > 0.8 and gene significance > 0.3. Homologous genes in *Arabidopsis thalian* (TAIR10) were identified using NCBI blastp with a *p*-value cutoff of 1 × 10^−10^.

### 4.7. Calculation of Single-Sample Gene Set Enrichment Analysis (ssGSEA) Score of GO Terms

The normalized read counts of leaf samples were used to calculate the ssGSEA score. The direct GO annotation for each gene were extracted from annotation data downloaded from the Phytozome website (https://phytozome-next.jgi.doe.gov/, accessed on 22 July 2023) and the indirect GO annotations were recovered using the “buildGOmap” function in R package “clusterProfiler” [40]. The ssGSEA score for each GO term in each leaf sample was calculated using using R package “GSVA” [27].

### 4.8. Identification of GO Terms with Differentially Expressed Profiles

The comparisons of ssGSEA scores of GO terms were conducted between ETW and all samples from hexaploid wheat (TAA10 and XX329) as well as between ETW and all samples from the other genotypes (TAA10, XX329, and TTR13). The comparisons were conducted using the R package “limma”, and the raw *p*-values were adjusted by the FDR method [41]. GO terms with an adjusted *p*-value < 0.05 were identified as differentially expressed terms.

### 4.9. Construction and Visualization of GO Network

GO term relationships were extracted from the core ontology data available from the Gene Ontology website (http://geneontology.org/docs/download-ontology/, accessed on 22 July 2023). The relationships were filtered to keep “is_a”, “negatively_regulates”, “positively_regulates”, and “regulates”. Only connected GO terms both were significantly differentially expressed were retained. The GO networks were visualized using CytoScape (version 3.9.1) and manually annotated using “AutoAnnotate” plugin [42,43].

## Figures and Tables

**Table 1:** Summary of DEGs identified in all pairwise compositions across the four wheat genotypes.

	Comparison	Upregulated	Downregulated	Total
Leaf	ETW vs. TTR13	7555	7782	15,337
	ETW vs. TAA10	5132	4429	9561
	ETW vs. XX329	6138	3609	9747
	TAA10 vs. TTR13	4435	4224	8659
	XX329 vs. TTR13	4599	6117	10,716
	XX329 vs. TAA10	2213	3293	5506
Root-tip	ETW vs. TTR13	2418	2157	4575
	ETW vs. TAA10	537	1521	2058
	ETW vs. XX329	553	1715	2268
	TAA10 vs. TTR13	2754	1548	4302
	XX329 vs. TTR13	2505	1378	3883
	XX329 vs. TAA10	310	130	440

**Table 2:** Summary of Pearson’s correlation coefficients and *p*-values among traits in ETW population (*p*-values are bracketed).

	Plant Height	Tiller Number per Plant	Seed Number per Plant	Spikelet Number per Plant
Tiller number per plant	0.494 (0.003)			
Seed number per plant	0.002 (0.990)	−0.058 (0.740)		
Spikelet number per plant	0.726 (<0.001)	0.578 (<0.001)	0.048 (0.785)	
Seed setting rate	−0.138 (0.428)	−0.192 (0.268)	0.944 (<0.001)	−0.127 (0.466)

## Data Availability

The RNAseq data for this study have been submitted to NCBI SRA database and can be found under the following accession number PRJNA1002614.

## Supplementary Materials

The following supporting information can be downloaded at: https://www.mdpi.com/article/10.3390/ijms241814037/s1.
