# Supplementary material for: Developmental Instability and Gene Dysregulation in an Extracted Tetraploid from Hexaploid Wheat
Source: Int J Mol Sci. 2023 Sep 13;24(18):14037. doi: 10.3390/ijms241814037 (PMC10531679; doi:10.3390/ijms241814037)

Figure S1. Venn diagram of up-regulated and down-regulated DEGs in ETW vs. TAA10 and ETW vs. XX329. (A) Venn diagram of DEGs detected in leaf tissue; (B) Venn diagram of DEGs detected in root-tip tissue.

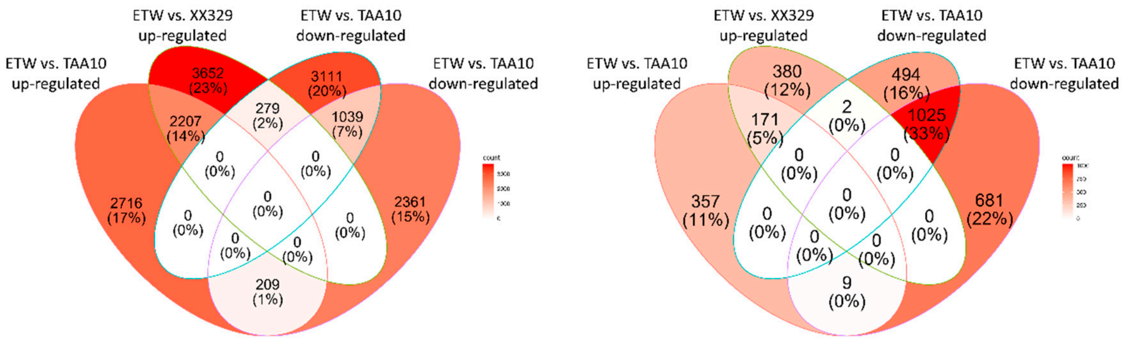

(A) (B)

Figure S2. Heatmap of the number of DEGs between different genotypes in leaf tissue.

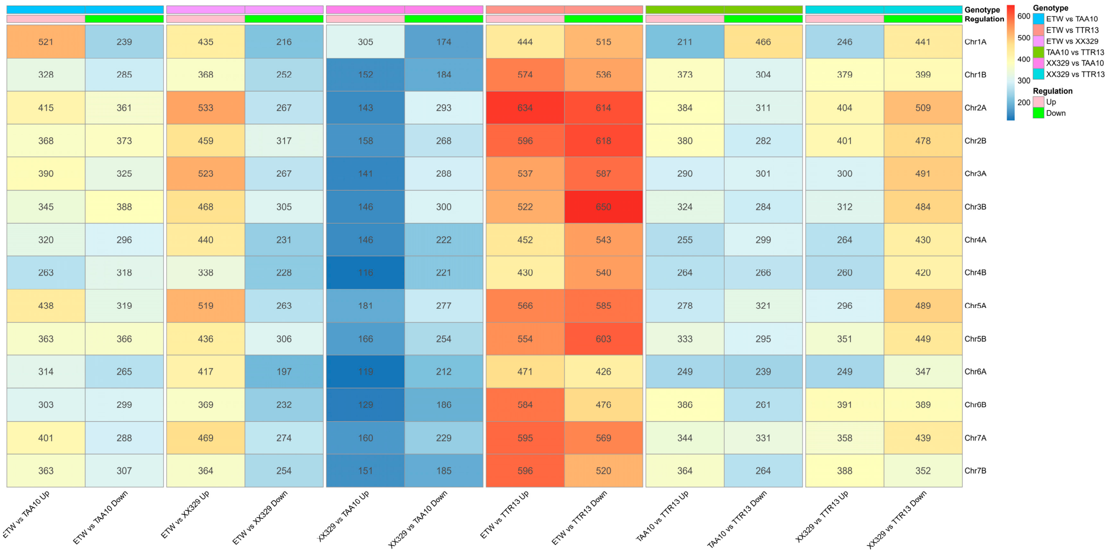

Figure S3. Heatmap of the number of DEGs between different genotypes in root-tip tissue.

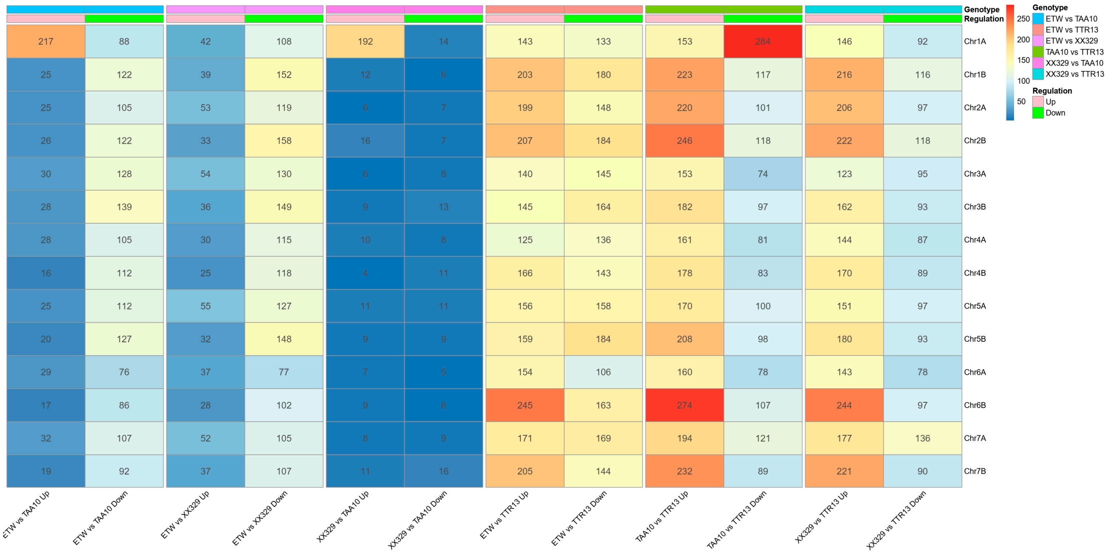

Figure S4. Distribution of DEGs along chromosome. Each vertical line represents a DEG. The color represents the log2 transferred fold-change between genotypes.

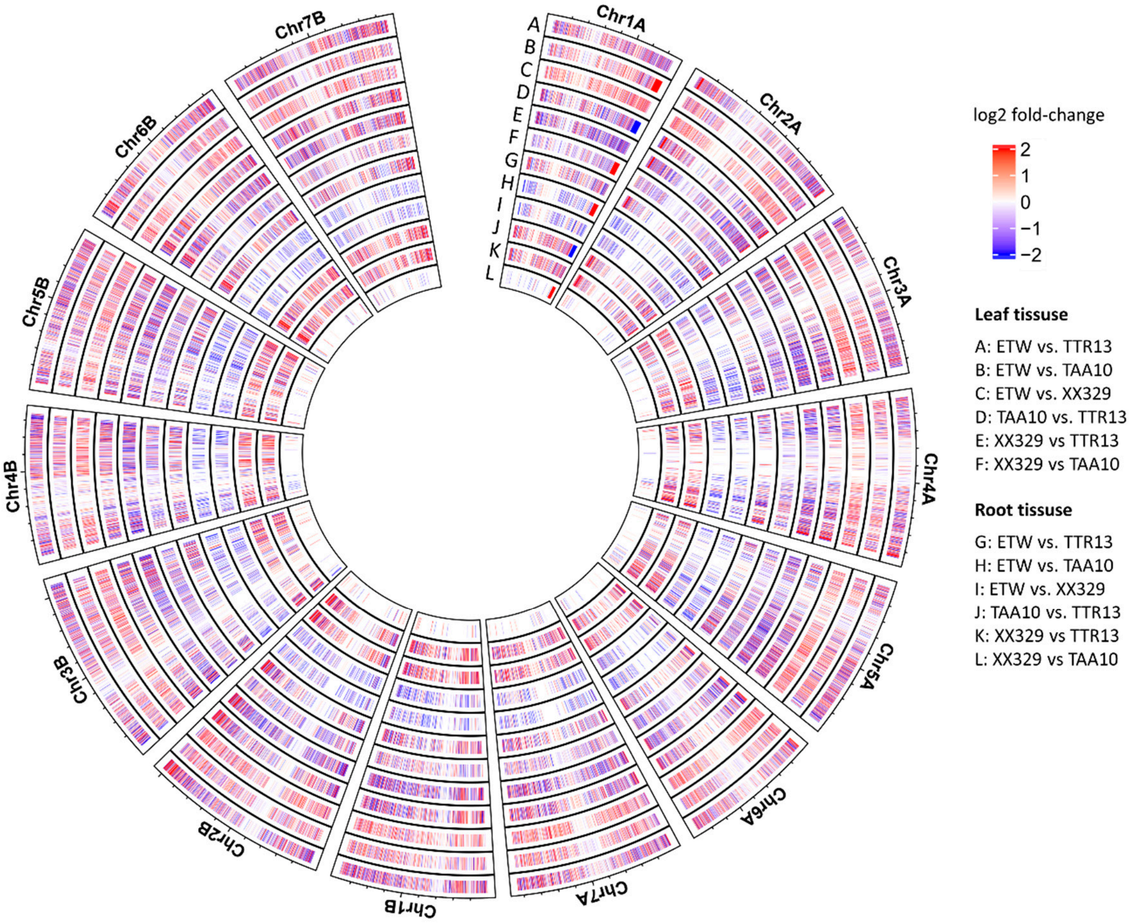

Figure S5. Over-represented GO terms in DEGs between genotypes. GO terms significantly (q-value < 0.05) were over-represented in DEGs are shown. The dot size and color correspond to proportions of DEGs the q-values.

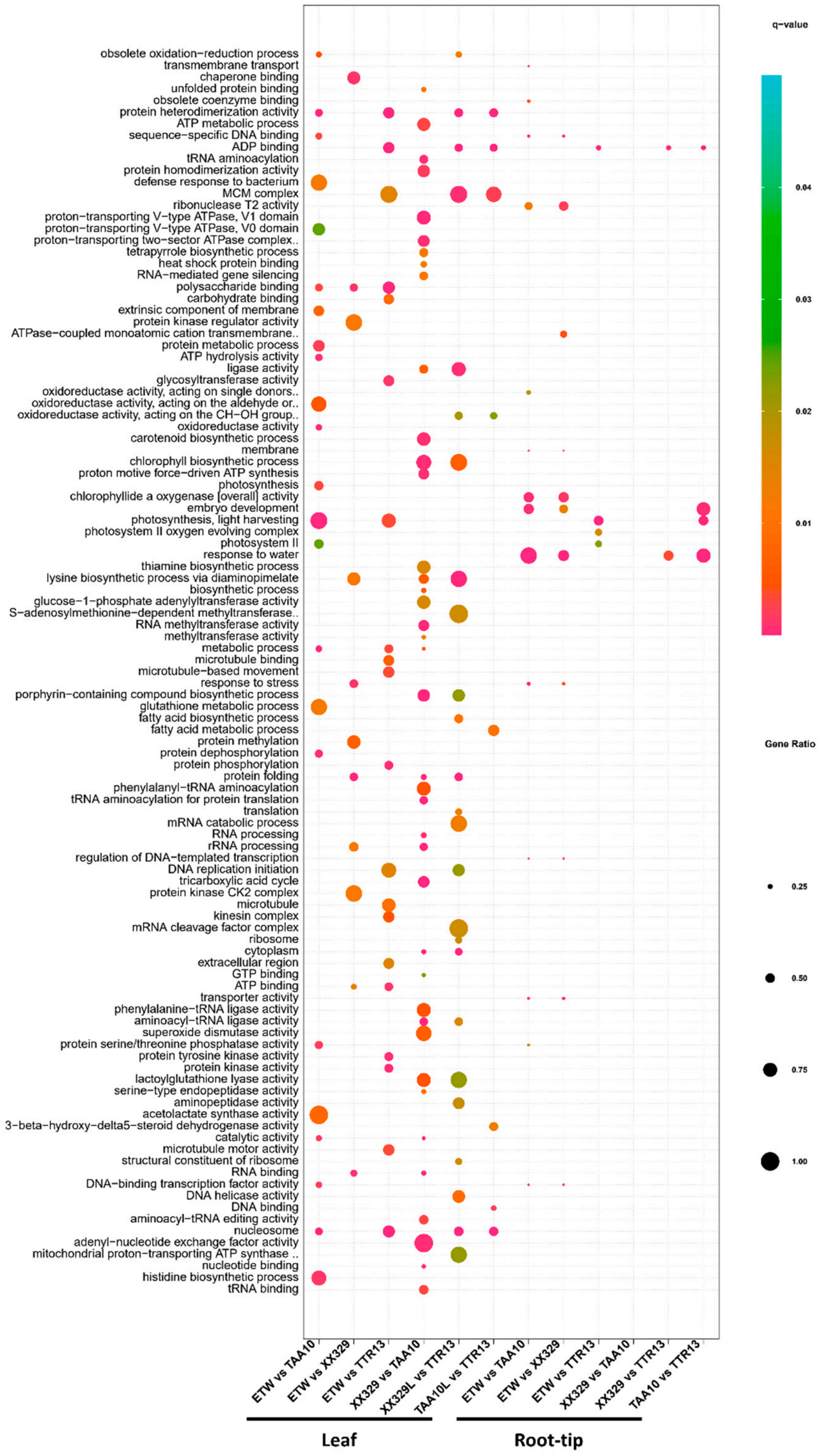

Figure S6. Over-represented Pfam terms in DEGs between genotypes. Pfam terms significantly (q-value < 0.05) were over-represented in DEGs are shown. The dot size and color correspond to proportions of DEGs the q-values.

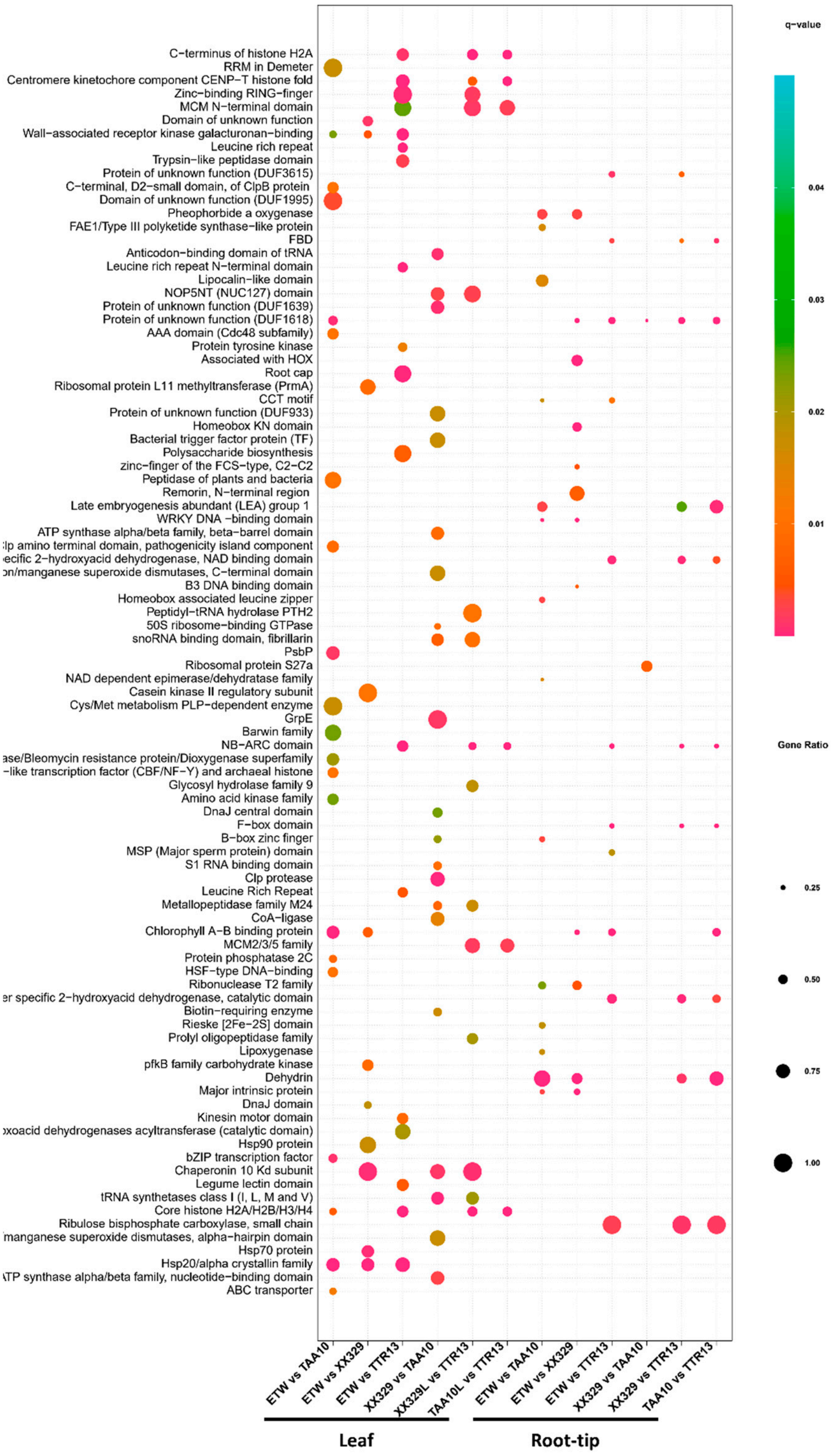

Figure S7. Network of significantly changed GO terms in the ETW population comparing to other genotypes. Each node represents a GO term. Edges represent relationships between terms. The log2 transferred fold change of ssGSEA sores between the population of ETW and those of hexaploids are shown in different colors.

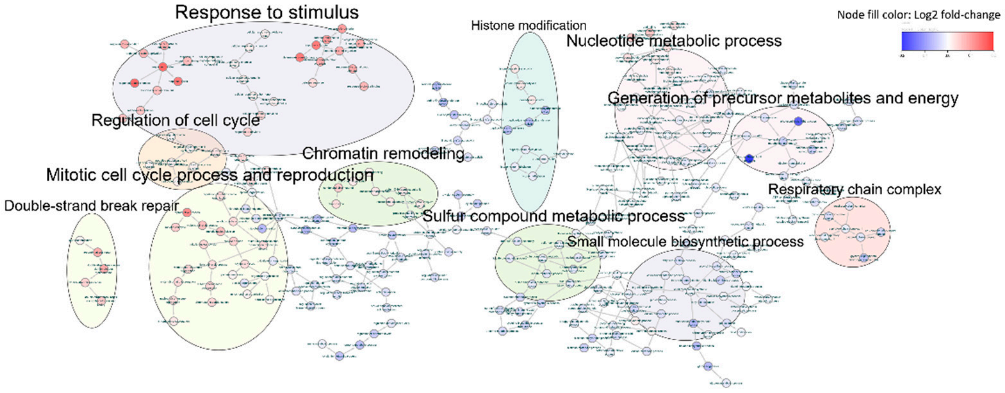

Supplement: Supplementary file 1 [file ijms-24-14037-s001.zip › Supplementary Figures.pdf]
