# Supplementary material for: Developmental Instability and Gene Dysregulation in an Extracted Tetraploid from Hexaploid Wheat
Source: Int J Mol Sci. 2023 Sep 13;24(18):14037. doi: 10.3390/ijms241814037 (PMC10531679; doi:10.3390/ijms241814037)
Supplement: Supplementary file 1 [file ijms-24-14037-s001.zip › Supplementary Tables.pdf]

Table S1. Descriptive statistics of different traits.

Traits	N[a]	Mean	Max	Min	Var[b]	Std[c]	Median	CV[d]	p.value[e]
**ETW**									
Plant height	30	43.33	56	32	32.64	5.71	43	13.18	0.97
Tiller number per plant	30	8.93	16	4	6.69	2.59	9	28.94	0.49
Seed number per plant	30	41.27	105	0	715.44	26.75	38.50	64.82	0.20
Spikelet number per plant	30	520.67	960	160	25901.33	160.94	540	30.91	0.65
Seed setting rate	30	0.07	0.15	0	0	0.04	0.08	52.78	0.87
**TAA10**									
Plant height	30	72.80	88	52	66.79	8.17	72	11.23	0.80
Tiller number per plant	30	8.87	14	5	5.29	2.30	8.50	25.94	0.12
Seed number per plant	30	531.53	779	308	16294.26	127.65	521.50	24.02	0.68
Spikelet number per plant	30	562.67	840	320	18909.06	137.51	568	24.44	0.66
Seed setting rate	30	0.95	0.99	0.88	0	0.03	0.95	3.15	0.11
**XX329**									
Plant height	30	72.03	82	59	46.31	6.81	72.50	9.45	0.22
Tiller number per plant	30	8.83	13	4	5.73	2.39	9	27.10	0.50
Seed number per plant	30	471.53	704	222	17340.46	131.68	483	27.93	0.41
Spikelet number per plant	30	511.87	780	240	20520.26	143.25	530	27.99	0.55
Seed setting rate	30	0.92	0.97	0.86	0	0.03	0.93	3.78	0.15
**TTR13**									
Plant height	30	101.10	119	87	58.58	7.65	100.50	7.57	0.79
Tiller number per plant	30	14.67	22	8	12.78	3.58	15	24.38	0.72
Seed number per plant	30	1069.97	1656	529	78016.86	279.31	1108	26.11	0.73
Spikelet number per plant	30	1104	1680	544	80771.31	284.20	1140	25.74	0.69
Seed setting rate	30	0.97	0.99	0.92	0	0.02	0.97	1.86	0.02

a, the number of measured individuals; b, variation; c, standard deviation; d, coefficient of variation; e, p-value of Shapiro-Wilk normality test.

Table S2. Summary of p-values of Mann-Whitney test between genotypes.

	ETW	TAA10	TTR13
**Plant height**			
**TAA10**	< 0.001		
**TTR13**	< 0.001	< 0.001	
**XX329**	< 0.001	0.711	< 0.001
**Tiller number per plant**			
**TAA10**	0.970		
**TTR13**	< 0.001	< 0.001	
**XX329**	0.970	0.970	< 0.001
**Seed number per plant**			
**TAA10**	< 0.001		
**TTR13**	< 0.001	< 0.001	
**XX329**	< 0.001	0.160	< 0.001
**Spikelet number per plant**			
**TAA10**	0.356		
**TTR13**	< 0.001	< 0.001	
**XX329**	0.882	0.356	< 0.001
**Seed setting rate**			
**TAA10**	< 0.001		
**TTR13**	< 0.001	0.002	
**XX329**	< 0.001	0.010	< 0.001

Table S3. Descriptive statistics of different traits of ETW population grown in greenhouse condition.

Traits	N[a]	Mean	Max	Min	Var[b]	Std[c]	Median	CV[d]	p.value[e]
**Plant height**	35	33.37	45.80	14.80	48.47	6.96	33.60	20.87	0.31
**Tiller number per plant**	35	3.14	6	2	0.95	0.97	3	31.01	< 0.001
**Seed number per plant**	35	0.66	6	0	1.41	1.19	0	180.59	< 0.001
**Spikelet number per plant**	35	36.63	80	8	302.06	17.38	34	47.45	< 0.001
**Seed setting rate**	35	2.01	16.70	0	12.52	3.54	0	175.90	< 0.001

a, the number of measured individuals; b, variation; c, standard deviation; d, coefficient of variation; e, p-value of Shapiro-Wilk normality test.

Table S4. P-values of modified signed-likelihood ratio test (SLRT) for equality of trait CVs between genotypes.

		ETW	TAA10	TTR13
Plant height	TAA10	0.402		
	TTR13	0.004	0.037	
	XX329	0.078	0.365	0.239
		ETW	TAA10	TTR13
Tiller number per plant	TAA10	0.607		
	TTR13	0.397	0.805	
	XX329	0.790	0.926	0.617
		ETW	TAA10	TTR13
Seed number per plant	TAA10	< 0.001		
	TTR13	< 0.001	0.707	
	XX329	< 0.001	0.459	0.782
		ETW	TAA10	TTR13
Spikelet number per plant	TAA10	0.244		
	TTR13	0.370	0.863	
	XX329	0.648	0.510	0.709
		ETW	TAA10	TTR13
Seed setting rate	TAA10	< 0.001		
	TTR13	< 0.001	0.005	
	XX329	< 0.001	0.326	< 0.001

Table S5. Pearson's correlation coefficients between traits and modules.

Module	Plant height	Tiller number per plant	Seed number per plant	Spikelet number per plant	Seed setting rate
white	0.07	-0.14	0.02	0.02	-0.04
darkorange	-0.12	-0.12	-0.10	0.10	-0.13
lightgreen	0.07	0.00	0.02	0.18	-0.04
darkgreen	-0.11	-0.29	-0.42	-0.35	-0.27
yellow	0.03	0.08	-0.42	0.00	-0.42
tan	0.03	0.14	0.08	0.03	0.16
blue	0.15	0.15	-0.40	0.04	-0.35
purple	0.45	0.28	-0.14	0.45	-0.21
darkgrey	0.01	0.22	0.06	0.07	0.00
grey60	0.24	0.04	-0.26	0.08	-0.22
pink	0.25	0.24	-0.12	0.33	-0.18
cyan	-0.30	-0.47	-0.03	-0.40	0.03
royalblue	-0.10	-0.23	-0.33	-0.25	-0.29
darkred	-0.52	-0.21	-0.14	-0.56	-0.01
magenta	-0.25	-0.21	-0.19	-0.40	-0.10
red	-0.27	-0.29	-0.19	-0.36	-0.14
brown	0.05	-0.19	0.08	0.03	0.06
green	0.12	0.15	-0.03	0.27	-0.12
greenyellow	0.48	0.13	0.12	0.52	0.00
darkturquoise	0.12	-0.29	0.09	0.08	0.05
lightyellow	0.13	0.04	0.17	0.21	0.14
turquoise	0.16	0.16	0.38	0.16	0.37
lightcyan	-0.40	-0.24	0.20	-0.38	0.35
salmon	-0.19	-0.04	0.29	-0.22	0.36
midnightblue	-0.28	0.23	0.23	0.05	0.19
black	-0.26	-0.03	0.69	-0.04	0.66
orange	-0.50	-0.15	0.41	-0.35	0.43
grey	0.23	0.04	-0.37	0.13	-0.40

Table S6. P-values of Pearson's correlation test between traits and modules.

Module	Plant height	Tiller number per plant	Seed number per plant	Spikelet number per plant	Seed setting rate
white	0.676	0.422	0.893	0.887	0.833
darkorange	0.475	0.502	0.554	0.572	0.453
lightgreen	0.696	0.987	0.931	0.308	0.810
darkgreen	0.536	0.087	0.012	0.042	0.111
yellow	0.852	0.634	0.012	0.979	0.013
tan	0.864	0.407	0.637	0.886	0.369
blue	0.389	0.382	0.018	0.826	0.037
purple	0.006	0.106	0.438	0.006	0.224
darkgrey	0.976	0.214	0.738	0.685	0.996
grey60	0.163	0.813	0.125	0.668	0.200
pink	0.148	0.173	0.489	0.050	0.290
cyan	0.085	0.004	0.861	0.017	0.844
royalblue	0.558	0.186	0.053	0.154	0.090
darkred	0.002	0.226	0.413	0.001	0.946
magenta	0.143	0.215	0.269	0.017	0.570
red	0.115	0.092	0.265	0.036	0.438
brown	0.794	0.279	0.645	0.860	0.738
green	0.477	0.375	0.885	0.114	0.491
greenyellow	0.003	0.472	0.501	0.002	0.981
darkturquoise	0.495	0.087	0.619	0.639	0.769
lightyellow	0.473	0.824	0.330	0.227	0.429
turquoise	0.372	0.366	0.023	0.370	0.028
lightcyan	0.016	0.173	0.247	0.024	0.038
salmon	0.282	0.809	0.089	0.198	0.034
midnightblue	0.104	0.178	0.189	0.793	0.274
black	0.126	0.851	0.000	0.800	0.000
orange	0.002	0.382	0.014	0.038	0.011
grey	0.188	0.839	0.031	0.463	0.018
